# Removal of impacted esophageal foreign bodies with a dual-channel endoscope: 19 cases

**DOI:** 10.3892/etm.2013.1085

**Published:** 2013-04-29

**Authors:** CHANGXIONG WANG, PING CHEN

**Affiliations:** Digestive Endoscope Center, The Sixth Affiliated College of Wenzhou Medical College, People’s Hospital of Lishui City, Lishui, Zhejiang 323000, P.R. China

**Keywords:** dual-channel endoscope, impacted foreign bodies, esophagus, balloon catheter

## Abstract

There have been few reports concerning the endoscopic removal of impacted esophageal foreign bodies from patients. The objective of this study was to evaluate the effectiveness of dual-channel endoscopy in managing foreign-body ingestions in patients. A total of 19 patients with foreign-body ingestions between September 2008 and July 2011 were selected from the Digestive Endoscope Center in Lishui, a typical middle-sized city in China. The patients underwent endoscopy following admission. The impacted foreign bodies were successfully removed from 18 patients without complications using a dual-channel endoscope. One patient underwent surgery for an ingested denture following the failure of the endoscopic removal method. This study demonstrates that dual-channel endoscopic management may be a useful option for removing ingested foreign bodies from the esophagus.

## Introduction

Foreign-body ingestion is a commonly encountered problem in the clinic. Although the majority of foreign bodies ingested pass spontaneously through the gastrointestinal tract, foreign bodies become lodged in the esophagus in ∼10–20% of cases, which require immediate intervention, typically by non-surgical endoscopic management (1). When esophageal foreign bodies are impacted for longer than 24 h, complications, including esophageal perforation, esophageal fistula, aortoesophageal fistula and mediastinal abscesses, may occur, and mucosal edema or anabrosis around the wounded areas may complicate or even prevent the endoscopic removal of foreign bodies.

Foreign-body ingestion is a commonly encountered clinical problem in China. However, only a few studies concerning the endoscopic removal of esophageal foreign bodies from Chinese patients are available in the literature (2). In addition, in the majority of these studies the standard flexible endoscope, which has limitations in removing sharp or larger foreign bodies, was generally used for management in these patients. The dual-channel endoscope has two advantages over the single-channel endoscope. The dual-channel endoscope is slender and flexible, and has a larger inner diameter that expands the esophageal wall thereby protecting the mucosa, while the single channel endoscope is stiff. The dual-channel endoscope also allows the insertion of a balloon catheter with a pincer, which may increase the success rate of foreign-body removal while minimizing complications.

In the present study, we report our experience and the outcomes achieved by using a more powerful dual-channel endoscope to remove impacted esophageal foreign bodies from 19 Chinese patients admitted to the Digestive Endoscope Center (Lishui, China) between September 2008 and July 2011. Our study suggests that dual-channel endoscopic management may be the optimal choice for removing impacted esophageal foreign bodies, including those that are large and/or sharp, from patients over a broad range of ages.

## Patients and methods

### Patients

Among 19 patients with impacted foreign bodies admitted to the People’s Hospital of Lishui City (Lishui, China), 13 were male and 6 were female, with ages ranging from 7 to 78 years (Table I). The impacted foreign bodies were identified at different locations with 11 cases in the upper esophagus, 5 cases in the middle and 3 cases in the lower esophagus. The delay time between ingestion and initial examination varied from 2 to 18 h and the endoscopic interventions were performed within 24 h. The types of foreign bodies found in these cases included mammalian bones (6 cases), peach kernels (2 cases), a jujube kernel (1 case), a denture (1 case), fish bones (3 cases), encapsulated pills (1 case), toothpicks (2 cases), a key (1 case), a toy (1 case) and a beer cap (1 case).

### Endoscopic management

A dual-channel endoscope (Fujinon EG-250D5; Fujifilm (China) Investment Co., Ltd. Pudong New Area, Shanghai, China) was used to remove the impacted foreign bodies. Based on the shapes and properties of the foreign bodies, suitable accessories were chosen, including rat-toothed forceps, an alligator clamp, snare, trielcon, basket and 2.8-mm biopsy forceps. A Boston CRE™ balloon dilatation catheter (Model Number: M00558380; Boston Scientific, Raffles city, Shanghai, China) with a diameter of 6–15 mm was used for the majority of cases.

## Results

### Initial management

The patients were closely monitored for symptoms, including subcutaneous emphysema, cough and neck tenderness prior to endoscopic management. X-ray and CT scanning methods were used to identify foreign-body shapes, and monitor the impacted areas and adjacent tissues and organs for possible complications such as esophageal inflammation, perforation and fistula. Cardiothoracic surgeons were consulted for potential risks and written consent forms were obtained from the patients or their guardians after they had been informed of the planned treatment.

### Endoscopic management

**Figure 1.**
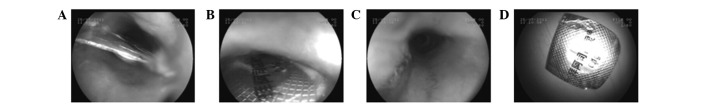
Images of the endoscopic management process for the removal of an encapsulated pill from a patient’s esophagus. (A) Shows a medication (aspirin tablet) with an outer plastic cover with both sides impacted in the esophageal cavity. (B) Shows the foreign body (reverse of the outer plastic cover) as viewed through the endoscope. (C) Shows the wound in the esophageal wall caused by the impacted medication following removal. (D) Shows that the medication and its plastic cover were removed successfully.

Each patient received continuous low-flow oxygen inhalation through nasal catheters and intravenous injection of propofol (1.5–2.0 mg/kg). When the patient were under anesthesia with no eyelash reflex, the endoscope was used to intubate the esophagus to locate the foreign body. The impacted area and damaged esophageal mucosa were closely examined prior to a balloon-tipped catheter being gently inserted and moved to a position 1.0–1.5 cm away from the impacted foreign body. The inner diameter of the right side clamp channel of the dual-channel endoscope was larger than the channel of a single-channel endoscope. The balloon was inserted via the channel of the right side clamp while the left side was used to insert other accessories, namely rat-toothed forceps, to aid in the foreign-body removal. In general, one side of the foreign body detached when the esophagus was expanded following balloon inflation. The appropriate accessory was then used to clamp the detached part of the foreign body, which was then removed gently. The endoscope, the foreign body and the inserted device were slowly removed simultaneously. During the procedure, efforts were made to minimize esophageal mucosal damage. An example of the removal of an encapsulated pill is shown in Fig. 1.

Following successful foreign-body removal, the affected areas were routinely checked with the endoscope for any mucosal damage, bleeding, residual foreign bodies, perforations and complicating diseases, including pharyngalgia and subcutaneous emphysema. In addition, antibiotics and other necessary medications were administered to the patients following endoscopy.

### Outcomes of endoscopic management

Foreign bodies were successfully removed in 18 cases using the dual-channel endoscope. Among these 18 cases, two foreign bodies had sizes (length or diameter) >12 cm and one foreign body had perforated the esophagus, and so were considered to be difficult to remove using a standard flexible endoscope. In addition, no complications were observed in our dual-channel endoscopic management. Dual-channel endoscopic removal failed in only one patient in whom the two ends of a denture (∼30×25 mm size) had perforated into the middle esophagus ∼25 cm away from the incisors. The foreign body did not separate from the mucosa following balloon expansion at the esophagus. Endoscopic management was finally abandoned due to the risk of heavy bleeding from the aortic arch to which the foreign body was adjacent, and the patient was then subjected to surgical removal.

## Discussion

Esophageal perforation resulting from impacted foreign bodies is a serious hazard encountered in the clinic (3). However, there are no reported epidemiological data on esophageal foreign bodies in China, and only few studies have been published on esophageal foreign body impactions in Chinese patients (4,5). In the present study, we report our experience and the outcomes of the removal of foreign bodies using a dual-channel endoscope with the aid of a balloon catheter. The balloon catheter is useful for dual-channel endoscopic foreign-body management as the inflated balloon causes the esophageal wall to expand, following which one side of the foreign body often detaches from the esophagus. This then facilitates clamping the foreign body for removal without further damage to the mucosa. In addition, the balloon catheter method also greatly reduces the risk of heavy bleeding or esophageal perforation following foreign-body removal as the inflated balloon suppresses esophageal bleeding while emergency surgery is in progress. We observed that the optimal location of the inserted balloon in the esophagus is ∼1.0–1.5-cm away from the far end of the endoscope. If the balloon is too close to the foreign body, it may worsen the condition by pressing the foreign body into the esophageal walls or the balloon may be perforated by a sharp foreign body.

The dual-channel endoscope has certain advantages over the standard flexible endoscope for foreign-body removal. A flexible endoscope that is generally 9–10 mm in diameter is not capable of removing a sharp or hooked foreign body or a larger foreign body (6–8). By contrast, the dual-channel endoscope has a greater diameter which greatly facilitates the removal of sharp or larger foreign bodies. In addition, a dual-channel endoscope may be used with a balloon catheter to minimize mucosal damage by expanding the esophagus. In contrast to a complication rate of ∼5% for standard flexible endoscopic management (2), our dual-channel endoscopic management for foreign-body removal in 19 Chinese patients aged between 7 and 78 years did not cause any complications in 18 patients. One patient presented with an impacted foreign body close to the main aortic arch and underwent surgery due to the risk of heavy bleeding. These results indicate that the dual-channel endoscope may prove to be a useful tool for removing ingested foreign bodies, particularly sharp or large foreign bodies, from the esophagus.

## Figures and Tables

**Table I.** Patient information and management methods.

	Age			Gender		Management	
		
	Child	Adult	Elderly	Male	Female	Endoscopy	Surgery
No. of patients	3	7	9	13	6	18	1

Children (<18 years), Adult (18–60 years), Elderly (>60 years).
